# Impact of ACCELERATE Paediatric Strategy Forums: a review of the value of multi-stakeholder meetings in oncology drug development: ACCELERATE in collaboration with the European Medicines Agency with participation of the Food and Drug Administration

**DOI:** 10.1093/jnci/djad239

**Published:** 2023-11-17

**Authors:** Andrew D J Pearson, Teresa de Rojas, Dominik Karres, Gregory Reaman, Nicole Scobie, Elizabeth Fox, Giovanni Lesa, Franca Ligas, Koen Norga, Karsten Nysom, Alberto Pappo, Brenda Weigel, Susan L Weiner, Gilles Vassal

**Affiliations:** ACCELERATE, Brussels, Belgium, Europe; ACCELERATE, Brussels, Belgium, Europe; Paediatric Medicines Office, Scientific Evidence Generation Department, Human Division, European Medicines Agency (EMA), Amsterdam, The Netherlands; US Food and Drug Administration (FDA), Silver Spring, MD, USA; Zoe4Life, Vaud, Sullens, Switzerland; St Jude Children’s Research Hospital, Memphis, TN, USA; Paediatric Medicines Office, Scientific Evidence Generation Department, Human Division, European Medicines Agency (EMA), Amsterdam, The Netherlands; Paediatric Medicines Office, Scientific Evidence Generation Department, Human Division, European Medicines Agency (EMA), Amsterdam, The Netherlands; Antwerp University Hospital, Antwerp, Belgium; Paediatric Committee of the European Medicines Agency, (EMA), Amsterdam, The Netherlands; Federal Agency for Medicines and Health Products, Brussels, Belgium; Righospitalet, Copenhagen, Denmark; St Jude Children’s Research Hospital, Memphis, TN, USA; University of Minnesota, Minneapolis, MN, USA; Children’s Cancer Cause, Washington, DC, USA; ACCELERATE, Brussels, Belgium, Europe; Gustave Roussy Cancer Centre, Paris, France

## Abstract

In a landscape of an increasing number of products and histology and age agnostic trials for rare patient cancer, prioritization of products is required. Paediatric Strategy Forums, organized by ACCELERATE and the European Medicines Agency with participation of the US Food and Drug Administration, are multi-stakeholder meetings that share information to best inform pediatric drug development strategies and subsequent clinical trial decisions. Academia, industry, regulators, and patient advocates are equal members, with patient advocates highlighting unmet needs of children and adolescents with cancer. The 11 Paediatric Strategy Forums since 2017 have made specific and general conclusions to accelerate drug development. Conclusions on product prioritization meetings, as well as global master protocols, have been outputs of these meetings. Forums have provided information for regulatory discussions and decisions by industry to facilitate development of high-priority products; for example, 62% of high-priority assets (agreed at a Forum) in contrast to 5% of those assets not considered high priority have been the subject of a Paediatric Investigational Plan or Written Request. Where there are multiple products of the same class, Forums have recommended a focused and sequential approach. Class prioritization resulted in an increase in waivers for non-prioritized B-cell products (44% to 75%) and a decrease in monotherapy trials, proposed in Paediatric Investigation Plans (PIP) submissions of checkpoint inhibitors from 53% to 19%. Strategy Forums could play a role in defining unmet medical needs. Multi-stakeholder forums, such as the Paediatric Strategy Forum, serve as a model to improve collaboration in the oncology drug development paradigm.

There remains an unmet need in children and adolescents with cancer for drugs with novel mechanisms of action that not only improve survival (1) but also reduce the acute and long-term burdens of therapy (2). The development of new anticancer drugs demands an integrated approach of all stakeholders—academia, industry, regulatory agencies, and patient advocates.

The milestone US Food and Drug Administration (FDA) Research to Accelerate Cures and Equity for Children (RACE), Act came into effect in 2020 (3). This law complements the revised class waiver list of the European Paediatric Regulation (4) and the proposed reform of the European Union (EU) Pharmaceutical Legislation (5). These new legislative initiatives greatly facilitate the evaluation of medicinal products in children and adolescents based on a science-driven, mechanism of action–based approach that fulfills the needs of children rather than being driven by the adult indication.

In a landscape of a mechanism of action approach to drug development, the large number of products currently available for adults and the limited size of the relevant pediatric population mandates prioritization of products. The international multi-stakeholder organization ACCELERATE (6,7), with the European Medicines Agency (EMA) and subsequently the FDA, has created multi-stakeholder Paediatric Strategy Forums (8-17).

The goal of Paediatric Strategy Forums is to evaluate science, facilitate dialogue, share information, foster prioritization, inform subsequent pediatric drug development and clinical trial strategies, and accelerate the introduction of the most promising safe and effective medicines into standard-of-care. In this way, novel drugs with a similar mechanism of action can be contextualized in a noncompetitive setting, such that resources are targeted and pediatric patients are not enrolled in suboptimal clinical studies unlikely to benefit them. Due to the relative rarity of childhood cancers, especially with molecularly defined subpopulations, global coordination is critical to accelerate drug development and alleviate feasibility issues.

This article describes the Paediatric Strategy Forums, assesses their impact on pediatric oncology drug development, and highlights their model features as having potential applicability not only in pediatric oncology but also in adults.

## Paediatric Strategy Forum model

The multi-stakeholder structure of the Forums is crucial, with each stakeholder of equal value. Regulators actively participate in the Forums, but no regulatory decisions are made. Patient advocates are key contributors and highlight unmet needs of children and adolescents with cancer; pharmaceutical companies present data and development concepts.

Suggested topics for future Forums are voted on at the annual ACCELERATE conference. The Forums are advertised and expressions of interest sought from the pharmaceutical industry (a condition of their participation is to present publicly available data), clinicians, patient advocates, and regulators. All patient advocates who submit an expression of interest are invited to attend, and recently there has been a dedicated preparatory call for advocates to provide some scientific background for the Paediatric Strategy Forum.

The Forums are generally held over 2 days, with an emphasis on facilitating discussion and science-based consensus. A scientific review of the current landscape and therapeutic needs is first presented by academic experts, followed by presentations of nonclinical and clinical information on products being developed by pharmaceutical companies. The Forums finish in patient advocate comments, a strategic discussion, and consensus conclusions. A summary (agreed to by all participants) is published on the EMA, FDA, and ACCELERATE websites and rapidly as an open-access paper (8-17). After a Forum, product rather than class prioritization may be required, and this is achieved by a prioritization meeting, held without the participation of regulators (18) (Table 1).

**Table 1.** Details of the Paediatric Strategy Forums^a^

Forum	Topic	Date	Venue	Prioritization/Update meetings	Participants	Continents of participants	Products discussed	Companies
First	ALK inhibitors (8)	30-31 Jan 2017	EMA London		46	Europe, North America	6	5
Second	Mature B cell malignancies (9)	13-14 Nov 2017	EMA London		74	Europe, North America	20	14
Third	Checkpoint inhibitors in combination (10)	5-6 Sept 2018	EMA London		68	Europe, North America	20	16
Fourth	AML (11)	11-12 Apr 2019	Rotterdam	FLT3, CD123 and Menin	71	Europe, North America, Asia, Australia	26	18
Fifth	Epigenetic modifiers (12)	23-24 Jan 2020	Philadelphia	Menin and Bromodomain and extra-terminal inhibitor	64	Europe, North America	16	12
Sixth	Second on ALK inhibitors (13)	14-15 Jan 2021	Virtual		73	Europe, North America	8	5
Seventh	CAR-T cells (14)	25-27 May 2021	Virtual		232	Europe, North America. Asia, Australia	13	11
Eighth	Multi-targeted kinase inhibitors in bone sarcomas (15)	30 Nov- 1 Dec 2021	Virtual		180	Europe, North America, Australia	8	8
Ninth	MAPK pathway (16)	28-29 March 20222	Virtual	FDA mini-symposium, with EMA participation on Functional Endpoints (Visual Acuity) for Low Grade Gliomas	206	Europe, North America Australia, South America, Asia	17	10
Tenth	DNA damage repair pathway inhibitors (17)	27-28 October 2022	EMA, Amsterdam	Biomarkers and ATR inhibitors	124 63 in person 61 virtual	Europe, United States, Canada, and Japan	15	6
Eleventh	PI3K, AKT, mTOR, and GSK3β pathway inhibitors	3 & 4 April 2023	Dana Farber Cancer Institute. USA		146 48 in person 98 virtual	Europe, North America, Africa	9	8

a ALK = Anaplastic lymphoma kinase; EMA = European Medicines Agency; AML = Acute myleoid leukemia; CAR = Chimeric Antigen Receptor;  MAPK = Mitogen-activated protein kinase; PI3-K = Phosphatidylinositol 3-kinase; mTOR = Mammalian target of rapamycin; GSK3β = Glycogen synthase kinase-3 beta.

Table 1 shows the details of each Forum. The 11 Forums included 1289 participants from Europe, North and South America, Australia, New Zealand, Asia, Mexico, and Africa; 43 companies; and 28 patient advocate organizations. Forty-one percent of participants were from academia, 24% industry, 6% patient advocates, and 29% regulators. In the future, two in-person Forums are planned each year, in Europe and in the United States, with virtual prioritization meetings.

### Summary of the outcomes of the first 11 Paediatric Strategy Forums

The first pilot Forum, held at the EMA in January 2017, focused on anaplastic lymphoma kinase (ALK) inhibition (8). It demonstrated that the approach was feasible and could be highly relevant for pediatric cancer drug development. It concluded that ALK inhibitors should be accessible to children with anaplastic large cell lymphoma and inflammatory myofibroblastic tumor and that there was a deficiency of clinical trials and regulatory submissions: their submission was strongly encouraged.

The second Forum on childhood mature B-cell malignancies (9) demonstrated that multi-stakeholder prioritization of classes of medicinal products was achievable. There was a consensus based on scientific rationale of the class of products considered to have the greatest probability of being beneficial for patients with relapsed disease. In addition, the need to develop a global industry-supported, academic-sponsored study with compounds from different pharmaceutical companies using a master protocol was identified.

The third Forum on checkpoint inhibitors determined that except for Hodgkin lymphoma, hypermutant pediatric tumors, and certain other situations, monotherapy with checkpoint inhibitors had very limited activity in childhood and adolescent malignancies (10). There was no scientific rationale for children to be enrolled in new monotherapy trials of additional checkpoint inhibitors with the same mechanism of action of agents already studied unless additional scientific knowledge became available. Finally, it highlighted the need for an international intercompany registry of pediatric-specific early and late adverse effects of immunotherapies (currently being established).

The fourth Forum, on acute myeloid leukemia (AML), resulted, for the first time, in two prioritization meetings—FLT3 inhibitors and CD123 antibodies; this produced a consensus of high-priority classes of compounds, based on available scientific evidence, and proposed a coordinated development with cooperative groups (11). It also suggested a global master protocol to allow drug evaluation of multiple treatment strategies. The fifth, on epigenetic modifiers (12), concluded that menin inhibitors should be moved rapidly into pediatric development; a prioritization meeting on BET inhibitors resolved that further clinical development of other pan-BET inhibitors in children should await the results of the first pediatric clinical trial (18).

The sixth Forum, again on ALK inhibitors, concluded that there were more clinical trials and regulatory submissions since the first Forum, ALK inhibitors should be included in front-line therapy of anaplastic large cell lymphoma (ALCL), the efficacy and safety of lorlatinib needed to be determined in front-line therapy in patients with neuroblastoma, and ALK inhibitors with very good central nervous system (CNS) penetration should be evaluated in CNS tumors with *ALK* fusions (13).

The seventh Forum, on chimeric antigen receptor (CAR) T-cells, identified the role and approaches relevant to pediatric acute lymphoblastic leukemia, B-cell non-Hodgkin lymphoma, relapsed and refractory Hodgkin lymphoma, AML, T-ALL, and solid tumors (14).

The eighth, on multi-targeted kinase inhibitors in bone sarcomas, resolved that those randomized studies, currently being planned or in progress, in front-line and relapse will inform the further development of this class of product (15). Moreover, understanding of relevant predictive biomarkers and tumor biology is critical.

The ninth, on mitogen-activated protein kinase (MAPK) pathway inhibitors, determined that 1) understanding specific tumor biology is crucial to develop the optimal combinations; 2) validated functional endpoints should be devised; and 3) long-term follow-up of patients receiving MAPK pathway inhibitors is particularly crucial in view of the prolonged administration (16).

The 10th Forum, on DNA damage response pathway inhibitors, concluded that combinations of poly-ADP ribose polymerase (PARP) inhibitors with ataxia telangiectasia and Rad3-related (ATR) inhibitors, antibody drug conjugates with DNA topoisomerase I inhibitor related warheads, or targeted radiotherapy warrant evaluation (17). Additional monotherapy trials of PARP inhibitors with the same mechanism of action are not recommended, and a further meeting on relevant biomarkers and a prioritization meeting on ATR inhibitors were held.

The 11th Forum, on phosphatidylinositol 3-kinase (PI3K), AKT, mammalian target of rapamycin (mTOR), and glycogen synthase kinase-3 beta (GSK3β) pathway inhibitors, made 4 conclusions: 1) mutation-specific, CNS-penetrant PI3-K inhibitors should be evaluated in children with diffuse midline glioma in a rational biological combination; 2) future trials of mTOR inhibitors in childhood cancer should not be conducted without very strong biological rationale and supportive preclinical data; 3) further preclinical clinical and evaluation of GSK3β inhibitors is required; and 4) even where there is an AKT mutation (∼0.1%), the role of AKT inhibitors in pediatric cancers remains unclear.

### Impact of the Paediatric Strategy Forums

#### Effect on regulatory submissions after Forums

##### B-cell medicinal products

The Forum (9) concluded prioritization was required, as many medicines were being developed. In view of their mode of action, clinicians proposed that CAR T-cells, T-cell engagers, and antibody drug conjugates (ADC) had the greatest probability of providing benefit in relapse. The expectation was that there would be an increase in scientifically justified waivers for B-cell products except CAR T-cells, T-cell engagers, or ADC.

After the Forum, a more targeted approach toward Paediatric Investigation Plans (PIP) agreements was seen, with several plans agreed on for CAR T-cells, T-cell engagers, or antibody drug conjugates (ADC). The percentage of relevant B-cell products granted waivers increased from 44% (12/27) before the Forum to 75% (27/36) after the Forum, and the number of PIPs decreased from 56% (15/27) to 25% (9/36) (Table 2).

**Table 2.** Effect on regulatory submissions to the European Medicines Agency (EMA) after Paediatric Strategy Forums on mature B-cell malignancies and checkpoint inhibitors in combination^a^

Time period	Number of products	PIP	Full waivers	Combination	PIPs for monotherapy
**B-cell products**					
July 2007 to November 2017	27	15/27 (56%)	12/27 (44%)		
December 2017 to June 2021	36	9/36 (25%)	27/36 (75%)		
**Immune checkpoint inhibitors**					
July 2007 - September 2018	15	13/15 (87%) –all broad conditions, including melanoma	2/15 (13%) (both narrow adult conditions)	5/13 (39%) combination development; no extrapolation	8 (53%)
October 2018 to August 2021	16	5/16 (31%)	11/16 (69%) (3 with broad conditions; 9 with narrow adult conditions)	2/5 (40%) (2 with full extrapolation	3 (19%)

a PIP = pediatric investigation plan.

In the United States, in line with the conclusions of the Forum, an agreement has been made with proposed plans for pediatric investigation of anti-CD19 ADC, CD19 CAR-T cell products, and CD3/CD20 bispecific T-cell Engager (BiTE)s, some of which are associated with deferrals pending preliminary efficacy/safety data in adults. Agreement with planned requests for full waivers has been reached for anti-CD20 monoclonal antibodies, PI3-K delta inhibitors, Bruton’s tyrosine-kinase inhibitors, and BCMA-related products.

#### Immune checkpoint inhibitors

This Forum resulted in a collective agreement that there was no scientific rationale for children to be enrolled in new monotherapy trials of checkpoint inhibitors with the same mechanism of action of agents already studied unless additional scientific knowledge supporting a different approach became available (10). In addition, studies of combination were encouraged.

After the Forum, there was a more focused agreement on PIPs with checkpoint combinations. The number of waivers granted by the Paedaitric Commitee (PDCO) of the EMA increased from 13% (2/15) to 69% (11/16); conversely, the number of PIPs decreased from 87% (13/15) to 31% (5/16). Two of the five PIPs were for combinations, and the number of PIPs for monotherapy decreased from eight (53%) to three (19%) (Table 2).

From 2019, the FDA has agreed to full waivers for single-agent pediatric assessments of 7 programmed death-1 (PD-1) or programmed death-1 ligand (PD-L1) inhibitors. Deferral of pediatric assessments of several bispecific or co-formulated antibodies against PD-1 and other immune checkpoint inhibitors (eg, cytotoxic T-lymphocyte–associated antigen 4 [CTLA-4], ligand lymphocyte activation gene-3 [LAG-3], T cell immunoreceptor with Ig and ITIM domains [TIGIT]) are being considered, pending demonstration of adult proof-of concept and improved efficacy in the same pediatric indications where activity of the PD-1/PD-L1 axis inhibition has been established.

#### Impact of Paediatric Strategy Forum ALK inhibition

Two Forums (2017 and 2021) focused on ALK inhibition (8,13), an important target in pediatric malignancies. At the time of the first Forum, there were no regulatory approvals in children. The Forum concluded that obtaining these was of paramount importance and that the available academically generated data showing activity of crizotinib should be filed. In the 4-year interval between the two Paediatric Strategy Forums, the number of open pediatric trials of ALK inhibitors increased from 30 to 13 (19), and there were regulatory submissions for crizotinib and brigatinib. The FDA approved crizotinib for the treatment of pediatric patients with relapsed or refractory anaplastic large-cell lymphoma (20), and there are ongoing PIPs for brigatinib and for crizotinib.

#### Current regulatory and trial status of products presented at Paediatric Strategy Forums

Overall, 34 of 75 (45%) products presented at the first 4 Forums were considered high priority, and of these 62% have been the subject of a PIP or Written Request; in contrast, only 5% of non-high-priority products progressed to a PIP or Written Request (Table 3 and Supplementary Table 1, available online). In addition, 65% of high-priority (but only 23% of non-high-priority) products now have an open clinical trial.

**Table 3.** Summary current regulatory and trial status of assets presented at the first 5 Paediatric Strategy Forums^a^

Forum	Products discussed	High priority—PIP or WR	Not high priority PIP or WR	High priority clinical trials	Not high priority clinical trials
ALK	6	2/4	0/2	4/4	1/2
B-cell	21	8/8^a^	0/14	6/8	4/14
Checkpoint	23	4/8	05/15	6/8	5/15
AML	25	7/14	2/11	6/14	4/11
Overall	75	21/34 (62%)	2/42 (5%)	22/34 (65%)	28/42 (33%)

a The consensus for Bruton's tyrosine kinase (BTK) inhibitors was that new additional trials should not commence until the results of the SPARKLE trial (NCT02703272) (36) were known especially in view of the very small numbers of available.  ALK = anaplastic lymphoma kinase; AML = acute myeloid leukemia; PIP = pediatric investigation plan; WR = written request.

#### Platform trials

At the Forum on mature B-cell malignancies, the many benefits of conducting industry-supported, academic-sponsored studies with compounds from different pharmaceutical companies were highlighted. Participants concluded that a master protocol in rare populations should be conducted to very high-quality standards with “intent to file,” and early input should be sought from regulators. As a result of this, Global Platform Study of Novel Medicines in Paediatric and Adolescent Relapsed and Refractory B-cell Non-Hodgkin Lymphoma (Glo-BNHL), a global platform clinical trial, was developed to assess the efficacy and safety of multiple prioritized novel agents for pediatric relapsed and refractory B-cell non-Hodgkin lymphoma. A robust predefined scientific prioritization process ensures that only those products showing the most promise are taken forward. The EMA, not part of Glo-BNHL, has provided Qualification Advice with a letter of support on its website on the methodological and scientific aspects, endorsing the trial (21).

Similarly, the fourth Forum recommended an industry-supported, academic-sponsored, “intent to file” global platform trial (Pediatric Acute Leukemia [PedAL]/European Paediatric acute myeloid leukaemia [EUpAL] Trial) (22) to allow evaluation of multiple products or combinations from different companies in pediatric AML within the same overall trial structure.

#### Perceptions of regulators and patient advocates

The EMA and European Commission Paediatric Action Plan has cited the Paediatric Strategy Forums as successful in conducting multi-stakeholder workshops in selected therapeutic areas (23,24). The FDA encouraged participation in international multi-stakeholder meetings, including the Paediatric Strategy Forums (25).

Since its founding, ACCELERATE has adopted the progressive practice of including patient advocates as equal partners in its organization and has openly sought advocates’ views in discussions and conclusions and as co-authors in publications. Advocates have strongly supported the Forums’ exclusive focus on meeting unmet medical needs in treating children with cancer and its approach on how to solve complex problems in pediatric oncology drug development. Over the course of 11 Forums, advocates and organizers have become more sophisticated about how advocates’ perspectives can add value to meeting outcomes. Early Forums included views that were typically concordant with those of researchers, while later meetings and publications set aside time and space in which advocates’ comments were separately summarized. Currently, all advocates attending the Forum are actively involved in the production of the “Patient Advocate’s Comments,” which are presented at the end of the Forum and are included in the published paper.

Advocates have emphasized that stakeholders should develop novel trial strategies to address the small numbers of patients for trials in some diseases and, in other cases, maximize the number of children in trials where unmet needs were great (eg, osteosarcoma). The inclusion of adolescents in adult trials, when scientifically indicated, was especially urged and was an example of families’ willingness to take research risks. Nonetheless, advocates consistently reminded stakeholders to take account of potential severe long-term effects as they consider emerging therapies. Advocates repeatedly urged that they be integrated very early in companies’ drug development planning so that advocates’ sense of urgency and real-world perspectives could benefit decision making.

#### Perceptions of industry and academic participants

Academic and industry participants in the first 4 Forums were invited to complete a questionnaire on the value of the Forums. Industry participants valued the presence of regulators and said that the Forums influenced the company’s pediatric applications (Table 4). Forums changed prioritization and selection of drugs for clinical trials of 62% of academic participants (Table 4).

**Table 4.** Perception of industry and academic participants

Question	N (%)
**Industry**	
Were the discussion/conclusions scientifically sound?	34/34 (100)
Did the presence of regulators provide added value?	34/34 (100)
Would you recommend other companies to participate in future Forums?	34/34 (100)
Was there an added value of the presence of regulators?	34/34 (100)
Did the Forum result in follow-up discussions in the company?	32/34 (94)
Were there actionable recommendations following the Forum?	29/34 (85)
Did the regulators’ comments influence your companies’ paediatric application?	27/34 (79)
Did the Forum result in change in your company’s decision?	18/34 (53)
Were there follow-up discussions with other meeting participants?	15/34 (44)
Did the Forum change plans for product development?	11/34 (32)
Did the Forum change the regulatory plans of the company for the relevant products?	7/34 (21)
**Academic**	
Did the Forum change prioritisation and selection of drugs for clinical trials?	26/42 (62)

## Discussion

Paediatric Strategy Forums arose out of the need to prioritize innovative new drugs in a rare disease environment where there will be more anticancer medicines available than can be evaluated in pediatric clinical trials. The Forums facilitate prioritization based on unmet medical needs and science. They have achieved this goal and have had a wider influence in the development of new anticancer drugs in children and adolescents (see Box 1). The key element for their success has been their multi-stakeholder nature, with each stakeholder being valued as an equal partner, especially patient advocates. The strong support of the EMA and the FDA as an opportunity for open discussion has been critical in their successful evolution.

Box 1.—General principles concluded during the eighth Paediatric Strategy Forum

- Pediatric oncology drug development should be science driven and should address children and adolescents’ unmet medical needs
- Prioritization is needed as not all oncology products in development in adults can be developed in children
- Global collaboration in both preclinical and clinical investigations is critical
- There should be early academia-multi-company engagement
- Very early involvement of regulators is essential when devising a development plan and a clinical trial
- Industry-supported, academic-sponsored international platform trials that provide clinical trial data that can be used for licensing purposes fit for filing are highly valuable
- International simultaneous regulatory submissions with a request for discussions at cluster calls are fully endorsed (31-34)
- Alignment is required between the scientific, regulatory, and Health Technology Assessment (HTA) (payers) requirements from the inception of a development plan and a clinical trial
- When there is strong preclinical evidence for the activity of a class of medicinal products or a strong mechanistic rationale, but uncertainty about the potential utility in pediatrics, early clinical evaluation with detailed embedded correlative biology of a member of the class in pediatrics should be prioritized and rapidly executed
- Where there are multiple products of the same class, there should be a focused and sequential approach to clinical evaluation.
- Stepwise PIPs: an agreed PIP is a living document that can be modified and evolve in light of new evidence based on needs and robust science have been endorsed (35)
- For very rare malignancies with the same biology in adults and children, the development and regulatory pathway should be the same in children, adolescents, and adults
- A coordinated approach is required between companies and academic cooperative group initiatives within the global regulatory framework

The data suggest that Forums resulted in changes in pediatric development plans (eg, mature B-cell malignancies, checkpoint inhibitors, ALK inhibitors). Products with high potential have been identified for accelerated development, in view of their biological rationale, strong preclinical activity, and ability to fulfill an unmet need. This is expected to reduce the current delay of a median of 6.5 years (26) in the evaluation of products in children compared to adults.

The Forums have been instrumental in facilitating collaboration among the pharmaceutical industry and the academic community, advocates, and regulators by providing a focus for interdisciplinary communication and exemplifying the key principle of shared responsibility between all stakeholders. They demonstrate that a consensus can be reached through multi-stakeholder discussion.

Outside pediatric oncology, Conect4children (c4c) (27), an Inonovative Medicines Initiative2 (IMI2) public and private project to create a EU sustainable pediatric clinical trial network, facilitating the development of medicines for the entire pediatric population, held its first successful multi-stakeholder meeting in April 2021 on pediatric inflammatory bowel disease (28). This meeting was based on the format of Paediatric Strategy Forums, and 3 other very successful meetings have now been held on atopic dermatitis (March 2022), medical devices (April 2023), and neonatal asphyxia (September 2023). As identified by the EMA, Paediatric Strategy Forums and these c4c meetings further demonstrate that multi-stakeholder conferences can identify and address therapeutic unmet needs and support strategic decision making (29). Finally, as identified by the EMA (30), the model of multi-stakeholder Paediatric Strategy Forums could be relevant in rare diseases and adult oncology.(EU) 

In conclusion, Paediatric Strategy Forums are a unique and effective modality in pediatric oncology. They define unmet medical needs and facilitate prioritization through multi-stakeholder dialogue and could be of value in the new EU Pharmaceutical Legislation (5). The Forums have been successful in prioritizing classes of medicinal products; however, their influence on prioritization of product appears less and requires detailed analysis. Furthermore, the remit of Paediatric Strategy Forums is being refined so that they can respond even better to the unmet needs of patients. For example, the 12th Forum will focus on diffuse midline glioma, a malignancy with a paucity of available therapeutic options. Experience has demonstrated that Forums held at an early stage in the drug development process are more effective so that class- and disease-specific developments can be harmonized and the landscape shaped. Paediatric Strategy Forums are continuously developing, evolving, and adapting to meet the changing needs of pediatric oncology drug development.

## Supplementary Material

djad239_Supplementary_Data

## Data Availability

This is a mini-review; no new scientific data was generated, and all data are within the paper.
