# Supplementary material for: Impact of ACCELERATE Paediatric Strategy Forums: a review of the value of multi-stakeholder meetings in oncology drug development: ACCELERATE in collaboration with the European Medicines Agency with participation of the Food and Drug Administration
Source: J Natl Cancer Inst. 2023 Nov 17;116(2):200–7. doi: 10.1093/jnci/djad239 (PMC10852613; doi:10.1093/jnci/djad239)
Supplement: djad239_Supplementary_Data [file djad239_supplementary_data.pdf]

Supplementary Table 1

Medicinal Product	Forum	High Priority for development	Relevant trials AFTER the Forum			EMA			FDA
			NCT number	Title	Phase, Start	Status, Date	EMA Ref	Indication of the PIP	Written Request, Date
Crizotinib	ALK	X	NCT03126916<br><br>NCT03874273	Iobenguane I-131 or Crizotinib and Standard Therapy in Treating Younger Patients With Newly-Diagnosed High-Risk Neuroblastoma or ganglioneuroblastoma<br><br>Study of Crizotinib in Children and Adolescents With Myofibroblastic Tumours	Phase 3, 2018<br><br>Phase 2-3, 2019, Russia	Modified PIP Jan/21	EMEA-001493-PIP03-18-M01	Treatment of paediatric patients with relapsed/refractory systemic ALK-positive ALCL<br><br>Treatment of paediatric patients with unresectable or relapsed/refractory ALK-positive IMT	Yes Nov11
Ceritinib	ALK	X	NCT02729961	Ceritinib With Brentuximab Vedotin in Treating Patients With ALK-Positive Anaplastic Large Cell Lymphoma	Phase 1-2, 2018	No PIP/W	N/A	N/A	No
Entrectinib	ALK		NCT04589845	Tumour-Agnostic Precision Immuno-Oncology and Somatic Targeting Rational for You (TAPISTRY) Platform Study	Phase 2, 2021	Modified PIP (Jan/19)	EMEA-002096-PIP01-16-M02	Treatment of NTRK fusion-positive locally advanced or metastatic solid tumours in paediatric patients from birth to less than 18 years of age who have either progressed following prior therapies or who have no acceptable standard therapies.	Yes Apr17
Lorlatinib	ALK	X	NCT03107988	NANT 2015-02: A Phase 1 Study of Lorlatinib (PF-06463922)	Phase 1, 2017	Waiver (Jan/21) for lung cancer	N/A	N/A	No
Alectinib	ALK		NCT04589845<br><br>NCT03194893	Tumour-Agnostic Precision Immuno-Oncology and Somatic Targeting Rational for You (TAPISTRY) Platform Study<br><br>A Roll Over Study of Alectinib in Patients With Anaplastic Lymphoma Kinase (ALK)-Positive or Rearranged During Transfection (RET)-Positive Cancer	Phase 2, 2021<br><br>Phase 3, 2017	Waiver (Dec/18) for lung cancer	N/A	N/A	No
Brigatinib	ALK	X	NCT04925609<br><br>NCT04260009	Brigatinib in Paediatric and Young Adult Patients With ALK+ ALCL, IMT or Other Solid Tumours<br><br>Pharmacokinetics, Safety, and Efficacy of Brigatinib Monotherapy in Paediatric and Young Adult Participants With ALK+ Anaplastic Large Cell Lymphoma, Inflammatory Myofibroblastic Tumours or Other Solid Tumours	Phase 1-2, 2021<br><br>Phase 1-2, 2020	Modified PIP (Dec/20)	EMEA-002296-PIP01-17-M02	Treatment in combination with standard chemotherapy in paediatric patients from 1 year of age and older with newly diagnosed ALK+ ALCL at high risk for recurrence	No

Polatuzumab vedotin - Roche	B-cell	NP	NCT04491370	Autologous Stem Cell Transplant Followed by Polatuzumab Vedotin in Patients With B-cell Non-Hodgkin and Hodgkin Lymphoma	Phase 1-2, 2021	Waiver Apr 18 for mature B-cell	N/A	N/A	No
Debio 1562 - Debiopharm International SA	B-Cell	NP	N/A	None found	N/A	Naratuximab, not in EMA database	N/A	N/A	No
CTL019 – Tivartis	B-Cell	X	NCT04225676<br><br>NCT04156659<br><br>NCT04094311<br><br>NCT03876769<br><br>NCT03610724	Study of Efficacy and Safety of Reinfusion of Tisagenlecleucel in Paediatric and Young Adult Patients With Acute Lymphoblastic Leukaemia (ALL)<br><br>Study of Tisagenlecleucel in Chinese Paediatric and Young Adult Subjects With Relapsed or Refractory B-cell ALL<br><br>Study of Out of Specification for Tisagenlecleucel<br><br>Study of Efficacy and Safety of Tisagenlecleucel in HR B-ALL EOC MRD Positive Patients<br><br>Phase II Open Label Trial to Determine Safety & Efficacy of Tisagenlecleucel in Paediatric Non-Hodgkin Lymphoma Patients	Phase 2, 2020<br><br>Phase 2, 2021<br><br>Phase 3, 2019<br><br>Phase 2, 2019<br><br>Phase 2, 2019	Tisagenlecleucel<br><br>Modified PIP Sep19	EMEA-001654-PIP02-17-M01	Paediatric patients with CD19+ relapsed or refractory mature B-cell non-Hodgkin's lymphoma	No
CD19 – KTE-C19 – Kite pharma	B-Cell	X	NCT05041309	Brexucabtagene<br><br>Long-term Follow-up Study for Participants of Kite-Sponsored Interventional Studies Treated With Gene-Modified Cells	Phase 2, 2021	KTE-X19<br><br>PIP Jan21	EMEA-001862-PIP03-20	Paediatric and adolescent subjects with relapsed or refractory B-cell non-Hodgkin lymphoma	No
Obinutuzumab - Roche	B-Cell	NP	N/A	None found	N/A	PIP Jan21<br><br>Waiver Dec 20 for glomerulonephritis	EMEA-001207-PIP02-19	Lupus nephritis (LN) in child onset systemic lupus erythematosus (cSLE)	No
CD3-CD19 – Blinitumomab – Amgen	B-Cell	X	NCT03849651<br><br>NCT03605589	TCRαβ-depleted Progenitor Cell Graft With Additional Memory T-cell DLI, Plus Selected Use of Blinatumomab, in Naive T-cell Depleted Haploidentical Donor Hematopoietc Cell Transplantation for Hematologic Malignancies<br><br>Pembro + Blina Combination in Paediatric and Young Adult Patients With Relapsed/Refractory Acute Leukaemia or Lymphoma	Phase 2, 2019<br><br>Phase 1, 2018	Blinatumomab<br><br>Modified PIP Apr20	EMEA-000574-PIP02-12-M03	Children with previously untreated high-risk first relapse of B precursor acute lymphoblastic leukaemia.	Yes Nov14
CD20 - CD3 TCB – RG6026 Roche	B-Cell	X	N/A	None found	N/A	CD20-TCB<br><br>PIP Mar20	EMEA-002648-PIP01-19	Children with relapsed or refractory high-grade mature B-cell non-Hodgkin lymphoma (B- NHL), including Burkitt lymphoma (BL), Burkitt leukaemia (mature B-cell acute	No

								lymphoblastic leukaemia FAB L3; B-AL), and diffuse large B-cell lymphoma (DLBCL)	
CD3 TBD - RG7828 - Roche	B-Cell	X	N/A	None found	N/A	CD20 CD3 TCB PIP Mar20	EMEA-002648-PIP01-19	Children with relapsed or refractory high-grade mature B-cell non-Hodgkin lymphoma (B- NHL), including Burkitt lymphoma (BL), Burkitt leukaemia (mature B-cell acute lymphoblastic leukaemia FAB L3; B-AL), and diffuse large B-cell lymphoma (DLBCL)	No
Pembrolizumab – Merck	B-Cell	XP	NCT03605589<br><br>NCT03445858<br><br>NCT03407144	Pembro + Blina Combination in Paediatric and Young Adult Patients With Relapsed/Refractory Acute Leukemias or Lymphoma<br><br>Pembrolizumab in Combination With Decitabine and Hypofractionated Index Lesion Radiation in Paediatrics and Young Adults<br><br>Safety and Efficacy of Pembrolizumab (MK-3475) in Children and Young Adults With Classical Hodgkin Lymphoma (MK-3475-667/KEYNOTE-667)	Phase 1, 2018<br><br>Phase 1, 2018<br><br>Phase 2, 2018	Modified PIP Jan18	EMEA-001474-PIP02-16-M01	Treatment of classical Hodgkin lymphoma with incomplete early response to front-line chemotherapy in children from 3 years to less than 18 years of age<br>- Treatment of relapsed or refractory classical Hodgkin lymphoma in children from 5 years to less than 18 years of age	No
Pembrolizumab - Merck	B-Cell	NP	=	=	=	Modified PIP Feb18	EMEA-001474-PIP01-13-M01	Treatment of advanced, untreated or previously treated, malignant melanoma in children from 12 year old to less than 18 years of age;<br>Treatment as monotherapy of a PD-L1 positive paediatric malignant solid tumour in children from 6 months to less than 18 years of age.	No
LAG-3<br>- BMS-986016<br>– BMS	B-Cell	NP	N/A	Relatlimab<br>None found for B-Cell	N/A	Not in the EMA db	N/A	N/A	No
Venetoclax<br>– AbbVie	B-Cell	NP	NCT04981912<br><br>NCT04161248	Rituximab + High-Dose Methylprednisolone Debulking Prior to Venetoclax for CLL & SLL Patients	Phase 1, 2021<br><br>Phase 1, 2019	Modified PIP Sep20	EMEA-002018-PIP02-	Treatment of relapsed or refractory Acute Lymphocytic Leukaemia (ALL)	Yes Jul18

			NCT03236857	Phase I Master Protocol of Novel Combination Therapy for Patients With Relapsed or Refractory Aggressive B-Cell Lymphoma<br><br>A Study of the Safety and Pharmacokinetics of Venetoclax in Paediatric and Young Adult Patients With Relapsed or Refractory Malignancies	Phase 1, 2017		16-M03	- Treatment of relapsed or refractory Acute Myeloid Leukaemia (AML)<br>Treatment of relapsed or refractory Non-Hodgkin lymphoma (NHL)<br>-Treatment of patients with relapsed or refractory neuroblastoma in patients from birth to less than 18 years of age	
Navitoclax<br>- AbbVie	B-Cell	NP	N/A	None found	N/A	Waiver Aug18 for myelofibrosis	N/A	N/A	No
BTK<br>- Ibrutinib – Janssen	B-Cell	X*	N/A	None found (for lymphoma)	N/A	Ibrutinib<br><br>Modified PIP Mar21	EMEA-001397-PIP03-14-M05	Treatment of children from 1 year to less than 18 years of age with newly-diagnosed and relapsed/ refractory mature B-cell lymphoma, that is, diffuse large B-cell lymphoma or Burkitt and Burkitt-like lymphoma	Yes May18
BTK<br>- Acalabrutinib (ACP-196)<br>- AZD	B-Cell	X*	NCT04546620	Acalabrutinib in Combination With R-CHOP for Previously Untreated Diffuse Large B-cell Lymphoma (DLBCL)	Phase 2, 2021	Acalabrutinib<br><br>Modified PIP Mar19	EMEA-001796-PIP03-16-M01	Treatment of children from 1 year to less than 18 years of age with newly-diagnosed and relapsed/ refractory mature B-cell lymphoma, that is, diffuse large B-cell lymphoma or Burkitt lymphoma or primary mediastinal lymphoma	No
ATR<br>- BAY1895344<br>– Bayer	B-Cell	NP	NCT05071209	Elimusertib<br><br>Elimusertib for the Treatment of Relapsed or Refractory Solid Tumours	Phase 1-2, 2021	BAY1895344<br><br>Not in EMA db	N/A	N/A	No
BET – BMS986158 – BMS	B-Cell	NP	NCT03936465	Study of the Bromodomain (BRD) and Extra-Terminal Domain (BET) Inhibitor BMS-986158 in Pediatric Cancer (Not relevant)	Phase 1, 2019	BMS986158<br><br>Not in EMA db	N/A	N/A	No
PI3-K<br>- Idelalisib<br>- Gilead	B-Cell	NP	NCT03349346	Idelalisib With Rituximab, Ifosfamide, Carboplatin, Etoposide (RICE) in Children and Adolescents	Phase 1, 2019	Waiver Nov18 for mature B-cell neoplasms	N/A	N/A	No
D22-TTC<br>- Bayer	B-Cell	NP	N/A	None found	N/A	D22-TTC	N/A	N/A	No

						Not in EMA db			
cc-122 and cc-220 – Celgene	B-Cell	NP	N/A	None found	N/A	cc-122 and cc-220<br><br>Not in EMA db	N/A	N/A	No
Pixantrone - Servier	B-Cell	NP	N/A	None found	N/A	Waiver Jul18 for NHL	N/A	N/A	No
Pembrolizumab Merck & Co	Checkpoint	X	NCT04995003<br><br>NCT04332874<br><br>NCT04171219<br><br>NCT04134559<br><br>NCT04118036<br><br>NCT03697824<br><br>NCT03605589	HER2 Chimeric Antigen Receptor (CAR) T Cells in Combination With Checkpoint Blockade in Patients With Advanced Sarcoma<br><br>A Study of Pembrolizumab Plus Local Chemotherapy Using Isolated Limb Infusion (ILI) for Patients With Sarcoma in the Arm or Leg<br><br>Talabostat and Pembrolizumab for the Treatment of Advanced Solid Cancers<br><br>Checkpoint Inhibition In Pediatric Hepatocellular Carcinoma<br><br>Abemaciclib + Pembrolizumab In Glioblastoma<br><br>Clinical Trial of Safety, Tolerability and Antitumor Activity of Genetically Engineered T Cells in Combination With Anti-Cancer Agents in Relapsed and Refractory Synovial Sarcoma Expressing New York Esophageal Antigen-1 (NY-ESO-1) and/or LAGE-1a<br><br>Pembro + Blina Combination in Paediatric and Young Adult Patients With Relapsed/Refractory Acute Leukaemia or Lymphoma	Phase 1, 2021<br><br>Phase 2, 2020<br><br>Phase 2, 2020<br><br>Phase 2, 2020<br><br>Phase 2, 2021<br><br>Phase 2, 2019<br><br>Phase 1, 2018	Modified PIP Feb18<br><br>Modified PIP Jan18	EMEA-001474-PIP01-13-M01<br><br>EMEA-001474-PIP02-16-M01	Treatment of advanced, untreated or previously treated, malignant melanoma in children from 12 years old to less than 18 years of age; Treatment as monotherapy of a PD-L1 positive paediatric malignant solid tumour in children from 6 months to less than 18 years of age.<br><br>Treatment of classical Hodgkin lymphoma with incomplete early response to front-line chemotherapy in children from 3 years to less than 18 years of age<br>Treatment of relapsed or refractory classical Hodgkin lymphoma in children from 5 years to less than 18 years of age	No
Avelumab Pfizer and Merck KgaA	Checkpoint	X	NCT05081180<br><br>NCT03617666	Study of Avelumab in Combination With Lenvatinib for Children With Primary CNS Tumours<br><br>Avelumab in the Frontline Treatment of Advanced Classical Hodgkin Lymphoma - a Window Study	Phase 1, 2021<br><br>Phase 2, 2019	Modified PIP Dec20	EMEA-001849-PIP02-15-M03	Treatment of paediatric patients from birth to less than 18 years old with a relapsed or refractory solid tumour or with a solid tumour as part of the first line treatment	No
Avelumab and axitinib Pfizer and Merck KgaA	Checkpoint	NP	N/A	Combination trial not found	N/A	Not in EMA db	N/A	N/A	Axitinib (NOT COMBINATION) YES, Aug20 (Avelumab no)
Radiotherapy Pembrolizumab, I131-MIBG,	Checkpoint	X	N/A	Combination trial not found	N/A	Not in EMA db	N/A	N/A	No

and anti-GD2d Minivan-Academic									
Cemiplimab Regeneron	Checkpoint	X	NCT03769181<br><br>NCT03690869	A Study of Isatuximab-based Therapy in Participants With Lymphoma<br><br>REGN2810 in Paediatric Patients With Relapsed, Refractory Solid, or Central Nervous System (CNS) Tumours and Safety and Efficacy of REGN2810 in Combination With Radiotherapy in Paediatric Patients With Newly Diagnosed or Recurrent Glioma	Phase 1-2, 2018<br><br>Phase 1-2, 2018	PIP Dec17	EMEA-002007-PIP02-17	Treatment of paediatric patients from birth to less than 18 years of age with a newly-diagnosed or recurrent high-grade glioma or with a newly-diagnosed diffuse intrinsic pontine glioma	Yes Dec18
Pembrolizumab/ dostarlimab and niraparib Tesaro	Checkpoint	XP	NCT04983745<br><br>NCT04544995	Niraparib and Dostarlimab in HRD Solid Tumours<br><br>Dose Escalation and Cohort Expansion Study of Niraparib and Dostarlimab in Paediatric Participants With Solid Tumours	Phase 2, 2021<br><br>Phase 1, 2020	Not in EMA db	N/A	N/A	Neither of the three
Tislelizumab and pamiparib BeiGene Inc. and Celgene	Checkpoint	X	N/A	Combination trial not found	N/A	Not in EMA db (tisle alone has waiver, pami too)	N/A	N/A	Neither of both
Nivolumab and entinostat – INFORM2 NivEnt Syndax Pharmaceuticals Academic dKiTZ sponsor	Checkpoint	X	NCT03838042	INFORM2 Study Uses Nivolumab and Entinostat in Children and Adolescents With High-risk Refractory Malignancies	Phase 1-2, 2019	Combination not in EMA db (entino has waiver)	N/A	N/A	Nivo Yes Sep14 (NOT combination)<br><br>Entinostat NO
Durvalumab And tremelimumab AstraZeneca	Checkpoint	NP	NCT03837899	Durvalumab and Tremelimumab for Paediatric Malignancies	Phase 1-2, 2019	Combination not in EMA db	N/A	N/A	Neither of both
Nivolumab and ipilimumab BMS and academic COG	Checkpoint	X	NCT04500548<br><br>NCT04416568<br><br>NCT04239040<br><br>NCT03668119	Testing the Combination of Two Immunotherapy Drugs (Nivolumab and Ipilimumab) in Children, Adolescent, and Young Adult Patients With Relapsed/Refractory Cancers That Have an Increased Number of Genetic Changes, The 3CI Study<br><br>Study of Nivolumab and Ipilimumab in Children and Young Adults With INI1-Negative Cancers<br><br>GVAX Plus Checkpoint Blockade in Neuroblastoma<br><br>A Study of Nivolumab Combined With Ipilimumab and Nivolumab Alone in Patients With Advanced or Metastatic Solid Tumours of High Tumour Mutational Burden (TMB-H)	Phase 1, 2021<br><br>Phase 2, 2020<br><br>Phase 1, 2020<br><br>Phase 2, 2018	Combination not in EMA db	N/A	N/A	Nivo yes Sep14<br><br>Ipi yes Jul14<br><br>(NOT combination)
Nivolumab and ipilimumab in CNS	Checkpoint	NP	NCT04323046	Immunotherapy (Nivolumab and Ipilimumab) Before and After Surgery for the Treatment of Recurrent or	Phase 1, 2020	Combination not in EMA db	N/A	N/A	Same

malignancies<br>BMS				Progressive High Grade Glioma in Children and Young Adults					
Nivolumab, ipilimumab and relatlimab (anti-LAG-3)<br>BMS	Checkpoint	NP	N/A	None found with this combination (there is one that is Nivo+Rela for chordoma)	N/A	Combination not in EMA db	N/A	N/A	Relatlimab NO
Nivolumab ipilimumab and NKTR-214<br><br>BMS	Checkpoint	NP	NCT04730349	Bempegaldesleukin<br><br>A Study of Bempegaldesleukin (BEMPEG: NKTR-214) in Combination With Nivolumab in Children, Adolescents and Young Adults With Recurrent or Treatment-resistant Cancer<br><br>(there are two other trials, for melanoma)	Phase 1-2, 2021	Combination not in EMA db	N/A	N/A	NKTR-214 NO
Anti-LAG-3<br>BI754091 (anti-PD-1) and BI 754111 (anti-LAG-3) monoclonal<br>Boehringer Ingelheim	Checkpoint	NP	NCT04138823<br><br>NCT03972150	A Study to Test Different Doses of BI 891065 Alone and in Combination With BI 754091 in Asian Patients With Different Types of Advanced Cancer (Solid Tumours)<br><br>A Study to Find the Best Dose of BI 836880 Alone and in Combination With BI 754091 in Japanese Patients With Different Types of Advanced Cancer	Phase 1, 2019<br><br>Phase 1, 2019	Not in EMA db	N/A	N/A	No
Anti-PD-1 and TSR-033 (TSR-033)<br>Tesaro	Checkpoint	NP	N/A	None found	N/A	Not in EMA db	N/A	N/A	No
Cemiplimab and bispecific CD20xCD3 antibody<br>Regeneron	Checkpoint	NP	N/A	Combination trial not found → The 2 trials with cemiplimab are listed above!<br><br>Also: there is one with isatuximab (see right below)	N/A	Combination not in EMA db	N/A	N/A	Cemi yes Dec18 (not combination)
Anti-PD-1 and isatuximab (CD38 monoclonal antibody)<br>Sanofi	Checkpoint	NP	NCT03860844<br><br>NCT03769181	Isatuximab in Combination With Chemotherapy in Paediatric Patients With Relapsed/Refractory Acute Lymphoblastic Leukaemia or Acute Myeloid Leukaemia<br><br>A Study of Isatuximab-based Therapy in Participants With Lymphoma	Phase 2, 2019<br><br>Phase 1-2, 2018	Isatuximab has modified PIP, but no combination	N/A	N/A	Lisa NO
Nivolumab and brentuximab bendamutisne, ipilimumab<br>BMS	Checkpoint	NP	N/A	Combination trial not found	N/A	Combination not in EMA db	N/A	N/A	Nivo yes Sep14<br><br>Brentuximab yes Jul21<br><br>(NOT combination)
M7824 (bifunctional fusion protein combining a PD-L1antibody and the extracellular domain of TGFbRII	Checkpoint	NP	N/A	None found	N/A	M7824 not in EMA db	N/A	N/A	No

neutralising TGFb)<br>EMD Serono									
Anti-PD-1 TSR 022 (anti-TIM-3)<br>Tesaro	Checkpoint	NP	N/A	None found	N/A	Not in EMA db	N/A	N/A	No
Cemiplimab<br>And<br>SAR439459<br>(anti TGF beta)<br>Sanofi	Checkpoint	NP	N/A	There is one (NCT04729725) but for >18y	N/A	Combination not in EMA db	N/A	N/A	Cemi yes Dec18<br><br>SAR439459 NO
Pembrolizumab and allogeneic cell therapy ilixadencel, ATMP<br>ImmunicumAB	Checkpoint	NP	N/A	None found	N/A	Combination not in EMA db	N/A	N/A	No
Pembrolizumab/<br>Atezolizumab and ATIMP<br>Autolus Ltd	Checkpoint	NP	N/A	None found	N/A	Combination not in EMA db	N/A	N/A	Pembro NO<br><br>Atezo yes Aug16
Midostaurin<br>Novartis Pharmaceutical Industry AG	AML	X	N/A	None found	N/A	Modified PIP Mar21	EMEA-000780-PIP01-09-M06	Treatment of paediatric patients with FLT3 mutated AML, newly diagnosed.	Yes May04
Gilteritinib<br>Astellas Pharma Global Development, Inc.	AML	X	NCT04293562<br><br><br><br><br>NCT04240002	A Study to Compare Standard Chemotherapy to Therapy With CPX-351 and/or Gilteritinib for Patients With Newly Diagnosed AML With or Without FLT3 Mutations<br><br>A Study of Gilteritinib (ASP2215) Combined With Chemotherapy in Children, Adolescents and Young Adults With FMS-like Tyrosine Kinase 3 (FLT3)/Internal Tandem Duplication (ITD) Positive Relapsed or Refractory Acute Myeloid Leukaemia (AML)	Phase 3, 2020<br><br><br><br><br>Phase 1-2, 2020	Modified PIP Mar21	EMEA-002064-PIP01-16-M03	Treatment of patients from 6 months to less than 18 years of age with relapsed or refractory FLT3/ITD positive acute myeloid leukaemia or newly-diagnosed FLT3/ITD positive acute myeloid leukaemia	Yes Jun20
IDH 1&2 Inhibitors<br>FORMA Therapeutics	AML	NP	??	??	??	??	??	??	??
Ivosidenib Agios	AML	X	N/A	Not for AML (NCT04195555)	N/A	PIP Sep18	EMEA-002247-PIP03-17	Treatment of paediatric patients from 2 to less than 18 years of age with newly diagnosed or relapsed or refractory acute myeloid leukaemia with an isocitrate dehydrogenase-1 mutation	No

Enasidenib Celgene	AML	X	NCT04203316	Enasidenib for the Treatment of Relapsed or Refractory Acute Myeloid Leukaemia Patients With an IDH2 Mutation	Phase 2, 2020	PIP Oct17	EMEA-001798-PIP02-16	Treatment of patients from 2 to less than 18 years of age with IDH2-mutated acute myeloid leukaemia	No
Flotetuzumab (bispecific CD123xCD3 DART) CD123<br><br>Laboratories Servier	AML	X	NCT04681105<br><br>NCT04158739<br><br>NCT03739606	Flotetuzumab for the Treatment of Relapsed or Refractory Advanced CD123-Positive Haematological Malignancies<br><br>Flotetuzumab for the Treatment of Paediatric Recurrent or Refractory Acute Myeloid Leukaemia<br><br>Flotetuzumab in Treating Patients With Recurrent or Refractory CD123 Positive Blood Cancer	Phase 1, 2020<br><br>Phase 1, 2020<br><br>Phase 2, 2020	Not in EMA db	N/A	N/A	No
XmAb®14045 (SQZ622) (bispecific CD123xCD3) Novartis Pharmaceutical Industry AG	AML	X	N/A	None found	N/A	Not in EMA db	N/A	N/A	No
SAR440324 (CD3xCD123 T cell engager) CD123 Sanofi	AML	X	N/A	None found	N/A	Not in EMA db	N/A	N/A	No
Gemtuzumab ozogamicin (ADC) CD33 Pfizer	AML	X	NCT04915612<br><br>NCT04793919<br><br>NCT04326439<br><br>NCT04293562	Liposomal Cytarabine, Daunorubicin, and Gemtuzumab Ozogamicin for the Treatment of Relapsed Refractory Paediatric Patients With Acute Myeloid Leukaemia<br><br>Treatment Study for Children and Adolescents With Acute Promyelocytic Leukaemia<br><br>AflacLL1901 (CHOA-AML)<br><br>A Study to Compare Standard Chemotherapy to Therapy With CPX-351 and/or Gilteritinib for Patients With Newly Diagnosed AML With or Without FLT3 Mutations	Phase 1, 2021<br><br>Phase 2, 2019<br><br>Phase 2, 2020<br><br>Phase 3, 2020	Modified PIP Oct17	EMEA-001733-PIP02-15-M01	Gemtuzumab ozogamicin is used in combination with induction regimens for the treatment of de novo and secondary newly-diagnosed acute myeloid leukaemia in paediatric patients aged 28 days up to less than 18 years	Yes Jan 01
AMG 330 & AMG 673 (BITE CD33) CD33 Amgen	AML	X	N/A	None found (1 for AMG 330 but >18y)	N/A	Not in EMA db (neither of them)	N/A	N/A	Neither of them
AMG 427 (BITE FLT3) FLT3 Amgen	AML	X	N/A	None found	N/A	Not in EMA db	N/A	N/A	No
Anetumab ravtansine (ADC) Mesothelin Bayer	AML	X	N/A	None found (1 for >18y)	N/A	Not in EMA db	N/A	N/A	No
Cusatuzumab (monoclonal antibody) CD70 Janssen	AML	NP	N/A	None found (4 for >18y)	N/A	Not in EMA db	N/A	N/A	No

Isatuximab (monoclonal antibody) CD38 Sanofi	AML	NP	NCT03860844	Isatuximab in Combination With Chemotherapy in Paediatric Patients With Relapsed/Refractory Acute Lymphoblastic Leukaemia or Acute Myeloid Leukaemia	Phase 2, 2019	Modified PIP May19	EMEA-002205-PIP01-17-M01	Treatment of relapsed, refractory and newly-diagnosed acute lymphoblastic leukaemia in combination with standard treatment in paediatric patients from 28 days to less than 18 years of age<br>Treatment of relapsed, refractory and newly-diagnosed acute myeloid leukaemia in combination with standard treatment in paediatric patients from 28 days to less than 18 years of age	No
MBG453 TIM-3 Novartis Pharmaceutical Industry AG	AML	NP	NCT04623216	Sabatolimab<br><br>Sabatolimab as a Treatment for Patients With Acute Myeloid Leukaemia and Presence of Measurable Residual Disease After Allogeneic Stem Cell Transplantation.	Phase 1-2, 2021	Not in EMA db	N/A	N/A	No
Nivolumab, ipilimumab with azacitidine PDL-1 BMS	AML	NP	NCT03825367	Not found with ipi, only nivo+aza:<br><br>Nivolumab in Combination With 5-azacytidine in Childhood Relapsed/Refractory AML	Phase 1-2, 2019	Not in EMA db	N/A	N/A	Nivo yes Sep14<br><br>Ipi yes Jul14<br><br>Aza No<br>(Not Combination)
Venetoclax BCL2 AbbVie jointly with Roche-Genentech	AML	X	??	??	??	Not in EMA db	N/A	N/A	Yes Jul18
Idasanutlin MDM2 Roche-Genentech	AML	NP	NCT04029688	A Study Evaluating the Safety, Tolerability, Pharmacokinetics and Preliminary Activity of Idasanutlin in Combination With Either Chemotherapy or Venetoclax in the Treatment of Paediatric and Young Adult Participants With Relapsed/Refractory Acute Leukemias or Solid Tumours	Phase 1-2, 2020	Modified PIP May20<br><br>- Modified PIP Mar20	EMEA-001489-PIP02-19<br><br>EMEA-001489-PIP01-13-M02	Treatment of children with a solid malignant tumour which is newly-diagnosed and metastatic, or refractory to first-line treatment<br>Treatment of children with first relapse of, or with frontline-refractory acute myeloid leukaemia	No
Siremadlin (HDM201) & MIK665/S6435 MDM2 and MCL- 1 Novartis	AML	NP	N/A	None found (1 trial with siremadlin, for >18y)	N/A	Combination not in EMA db	N/A	N/A	No

Pharmaceutical Industry AG									
BMS-986158 BET BMS	AML	NP	N/A	1 trial found (NCT03936465) but for solid tumours and lymphoma	N/A	BMS-986158<br><br>Not in EMA db	N/A	N/A	No
AMG 176 and AMG 397 MCL1 Amgen	AML	NP	N/A	None found	N/A	Not in EMA db	N/A	N/A	Neither of them
Alisertib Aurora A kinase Takeda Ltd	AML	NP	N/A	None found (3 for >18y)	N/A	Not in EMA db	N/A	N/A	No
Pevonedistat NEDD8-activating enzyme Takeda Ltd	AML	X	NCT03813147	Pevonedistat, Azacitidine, Fludarabine Phosphate, and Cytarabine in Treating Patients With Relapsed or Refractory Acute Myeloid Leukaemia or Myelodysplastic Syndrome	Phase 1, 2019	Modified PIP Mar18	EMEA-002117-PIP01-17-M01	Treatment of paediatric patients with newly diagnosed high risk AML or relapsed or refractory (R/R) AML	No
Pracinostat HDAC Helsinn HealthCare	AML	NP	N/A	None found (1 for >18y)	N/A	PIP Apr20	EMEA-002567-PIP01-19	Treatment, in combination with azacitidine, of children with relapsed acute myeloid leukaemia who are refractory to re-induction chemotherapy or who relapse for a second time	No
Vyxeos (CPX-351) Liposomal cytarabine and daunorubin<br><br>Jazz Pharmaceuticals	AML	X	NCT04915612<br><br>NCT04293562	Liposomal Cytarabine, Daunorubicin, and Gemtuzumab Ozogamicin for the Treatment of Relapsed Refractory Paediatric Patients With Acute Myeloid Leukaemia<br><br>A Study to Compare Standard Chemotherapy to Therapy With CPX-351 and/or Gilteritinib for Patients With Newly Diagnosed AML With or Without FLT3 Mutations	Phase 1, 2021<br><br>Phase 3, 2020	Cytarabine+ daunorubicin Modified PIP Aug19	EMEA-001858-PIP02-16-M03	Treatment of paediatric patients from 28 days to less than 18 years of age with relapsed/refractory acute myeloid leukaemia or in first relapse and with newly diagnosed acute myeloid leukaemia.	No

Supplementary Table 1 **- Current regulatory and trial status of assets presented at Paediatric Strategy Forums**

EMA db – https://www.ema.europa.eu/en/medicines/download-medicine-data#paediatric-investigation-plans-section[37]

PIP – Paediatric Investigational Plan; N/A - Not applicable
